# Early phenotypic detection of fluconazole- and anidulafungin-resistant *Candida glabrata* isolates

**DOI:** 10.1093/jac/dkac075

**Published:** 2022-03-22

**Authors:** Panagiota-Christina Georgiou, Maiken Cavling Arendrup, Joseph Meletiadis

**Affiliations:** Clinical Microbiology Laboratory, Attikon University Hospital, Athens, Greece; Unit of Mycology, Statens Serum Institute, Copenhagen, Denmark; Department of Clinical Microbiology, Rigshospitalet, Copenhagen, Denmark; Department of Clinical Medicine, University of Copenhagen, Copenhagen, Denmark; Clinical Microbiology Laboratory, Attikon University Hospital, Athens, Greece; Department of Medical Microbiology and Infectious Diseases, Erasmus MC, Rotterdam, The Netherlands

## Abstract

**Background:**

Increased fluconazole and echinocandin resistance in *Candida glabrata* requires prompt detection in routine settings. A phenotypic test based on the EUCAST E.DEF 7.3.2 protocol was developed for the detection of fluconazole- and anidulafungin-resistant isolates utilizing the colorimetric dye XTT.

**Methods:**

Thirty-one clinical *C. glabrata* isolates, 11 anidulafungin resistant and 14 fluconazole resistant, were tested. After optimization studies, 0.5–2.5 × 10^5^ cfu/mL of each isolate in RPMI 1640 + 2% d-glucose medium containing 100 mg/L XTT + 0.78 μΜ menadione and 0.06 mg/L anidulafungin (S breakpoint) or 16 mg/L fluconazole (I breakpoint) in 96-well flat-bottom microtitration plates were incubated at 37°C for 18 h; we also included drug-free wells. XTT absorbance was measured at 450 nm every 15 min. Differences between the drug-free and the drug-treated wells were assessed using Student’s *t*-test at different timepoints. ROC curves were used in order to identify the best timepoint and cut-off.

**Results:**

The XTT absorbance differences between fluconazole-containing and drug-free wells were significantly lower for the resistant isolates compared with susceptible increased exposure isolates (0.08 ± 0.05 versus 0.25 ± 0.06, respectively, *P *= 0.005) at 7.5 h, with a difference of <0.157 corresponding to 100% sensitivity and 94% specificity for detection of resistance. The XTT absorbance differences between anidulafungin-containing and drug-free wells were significantly lower for the resistant isolates compared with susceptible isolates (0.08 ± 0.07 versus 0.200 ± 0.03, respectively, *P *< 0.001) at 5 h, with a difference of <0.145 corresponding to 91% sensitivity and 100% specificity, irrespective of underlying mutations.

**Conclusions:**

A simple, cheap and fast phenotypic test was developed for detection of fluconazole- and anidulafungin-resistant *C. glabrata* isolates.

## Introduction

Invasive *Candida* infections are estimated to occur globally in more than 250* *000 patients per year^1^ and they are associated with high morbidity and mortality in immunocompromised patients, particularly in the ICU. Although *Candida albicans* is still the most frequent *Candida* species, *Candida glabrata* is the second to third most common *Candida* species, causing invasive candidiasis in the USA, Australia and Northern Europe.^1,2^ This is a challenge because *C. glabrata*, with its propensity to acquire azole^3,4^ and echinocandin resistance and up to 15% MDR phenotype, is difficult to treat.^2,5,6^

Given that the mortality of invasive candidiasis doubles with every day of delayed effective antifungal therapy, efforts have been made with regard to fast and accurate detection of resistance.^7^ The reference methods for antifungal susceptibility testing developed by CLSI^8^ and EUCAST^9^ require at least 24 h of incubation. Alternative tests have been proposed, based on molecular techniques,^10,11^ flow cytometry^12,13^ and MALDI-TOF MS,^14,15^ but they have limited application in routine laboratories because of multifactorial mechanisms of azole resistance,^16^ high complexity, lack of standardization and interpretative guidelines, increased cost and requirement for special equipment and skilled personnel.

Colorimetric methods have been used for antifungal susceptibility testing, since they generate clear-cut endpoints, based on detectable colour changes. XTT is a yellow, water-soluble tetrazolium salt that is rapidly reduced by mitochondrial dehydrogenases of metabolically active yeast cells.^17,18^ XTT-based methods have a wide use in antifungal susceptibility^19,20^ and biofilm^21^ studies. In the present study, we developed a phenotypic test based on the EUCAST E.DEF 7.3.2 protocol for the detection of fluconazole- and anidulafungin-resistant isolates utilizing the colorimetric dye XTT.

## Materials and methods

### Isolates

A total of 31 clinical *C. glabrata* isolates were used for both antifungal agents (Table 1), 11 anidulafungin resistant (R) (MICs 0.125–4 mg/L) and 20 anidulafungin susceptible (S) (MICs ≤0.008–0.06 mg/L) and 14 fluconazole resistant (R) (MICs >16 mg/L) and 17 fluconazole susceptible increased exposure (I) (MICs ≤16 mg/L). *Candida krusei* ATCC 6258 and *Candida parapsilosis* ATCC 22019 were used as quality control strains. All MICs were determined in triplicate using EUCAST E.DEF 7.3.2.^9^ Isolates were stored in 10% glycerol aqueous solution and were revived by subculturing onto Sabouraud glucose agar plates, 18–24 h prior.

**Table 1.** Median MICs and classification of each strain for anidulafungin and fluconazole

*C. glabrata* isolates	*FKS* mutation	Anidulafungin		Fluconazole	
		MIC (mg/L)	classification	MIC (mg/L)	classification
SSI-6133	*FKS2* D663E	0.25	R	32	R
SSI-2250	*FKS2* S663F	2	R	8	I
SSI-4857	*FKS2* L712-STOP/F659-DEL	4	R	>32	R
SSI-5324	*FKS2* F659-DEL	2	R	32	R
SSI-3203	*FKS1* S629P	2	R	>32	R
SSI-4843	*FKS1* L630Q + *FKS2* S663F	1	R	2	I
SSI-5128	*FKS2* S663P	2	R	>32	R
SSI-7033	*FKS2* l662W	0.5	R	>32	R
SSI-2696^a^	WT	0.125	R	>32	R
AUH-379	*FKS2* S663F	1	R	8	I
SSI-3775	*FKS2* F659S	2	R	16	I
SSI-7968	WT	0.016	S	>32	R
SSI-7966	WT	0.016	S	4	I
SSI-7967	WT	0.016	S	4	I
SSI-7969	WT	0.016	S	8	I
SSI-5502	WT	0.03	S	8	I
SSI-4914	*FKS2* F659L	0.06	S	2	I
SSI-64.37	WT	0.016	S	4	I
SSI-6244	WT	0.03	S	>32	R
SSI-6371	WT	0.03	S	32	R
SSI-62.64	WT	0.03	S	>32	R
SSI-61.31	WT	0.03	S	>32	R
SSI-61.10	WT	0.03	S	16	I
SSI-60.02	WT	0.016	S	16	I
SSI-7965	WT	0.016	S	16	I
SSI-4778	WT	0.03	S	>32	R
SSI-2717	WT	0.03	S	>32	R
AUH-249	WT	0.03	S	16	I
AUH-312	WT	0.016	S	16	I
AUH-1670	WT	0.016	S	16	I
AUH-1740	WT	0.03	S	16	I

S, susceptible; I, susceptible increased exposure; R, resistant; SSI, Statens Serum Institute; AUH, Attikon University Hospital.

a This *C. glabrata* isolate was cultured from a faeces sample from a patient with oesophagitis due to an MDR *C. albicans*. The *C. albicans* harboured an S645P alteration, whereas the *C. glabrata* had WT *FKS* genes but MICs spanning 0.06–0.125 mg/L.

### Compounds and medium

Pure powders of anidulafungin (Pfizer) and fluconazole (Sigma) were dissolved in DMSO to a concentration of 5 and 6.4 mg/mL, respectively. Stocks solutions were stored at −70°C until use. XTT (Sigma) was dissolved at a final concentration of 100–400 mg/L in water supplemented with menadione (Sigma) at a final concentration of 0.78–25 μM from a stock solution of 58 000 μM in absolute ethanol. RPMI 1640 medium (with l-glutamine; without bicarbonate) buffered to pH 7.0 with 0.165 M MOPS (Applichem and Sigma) with 2% d-glucose was used throughout the study.

### XTT susceptibility testing

For the XTT assay, 0.5–2.5 × 10^5^ cfu/mL of each isolate in RPMI 1640 + 2% d-glucose medium containing different concentrations of XTT/menadione and the antifungal drug were added to 96-well flat-bottom microtitration plates (200 μL/well). In order to find the optimal concentrations of XTT and menadione that differentiate resistant and susceptible isolates, 100–400 mg/L XTT with 0.78–25 μM menadione were tested together with anidulafungin and fluconazole concentrations close to the EUCAST breakpoints (0.12 and 0.06 mg/L anidulafungin and 32 and 16 mg/L fluconazole). Strains SSI-3203 (resistant to both antifungal drugs), SSI-7696 (susceptible to anidulafungin) and SSI-5502 (susceptible increased exposure to fluconazole) were used in optimization studies. Furthermore, addition of drugs 0, 2 and 4 h after inoculation was also assessed. Drug-free wells were also included. The plates were incubated at 37°C. The metabolic activity in each well was kinetically assessed by measuring the absorbance at 450 nm every 15 min for more than 18 h in a microplate reader (Tecan Infinite F200). Curves linked to metabolic activity were generated for each isolate and each condition.

### Statistical analysis

After subtraction of background absorbance, the differences in XTT absorbance between drug-free and drug-containing wells (deltaΧΤΤ-ABS) were calculated for each isolate and the differences between the resistant and the susceptible isolates were assessed with a non-parametric Mann–Whitney test at different timepoints for each antifungal drug. ROC curve analysis was used in order to determine the earliest timepoint with the highest area under the ROC (AUC_ROC_). Specificity and sensitivity in detecting resistant isolates were calculated at different timepoints and deltaXTT-ABS. From each timepoint the deltaXTT-ABS was chosen based on the highest likelihood ratio [sensitivity/(100−specificity)]. For deltaXTT-ABS with an undefined likelihood ratio (when specificity is 100%), the deltaXTT-ABS with the largest sensitivity was chosen. All analyses were performed using GraphPad Prism 5.03.

## Results

### Metabolic curves

**Figure 1.**
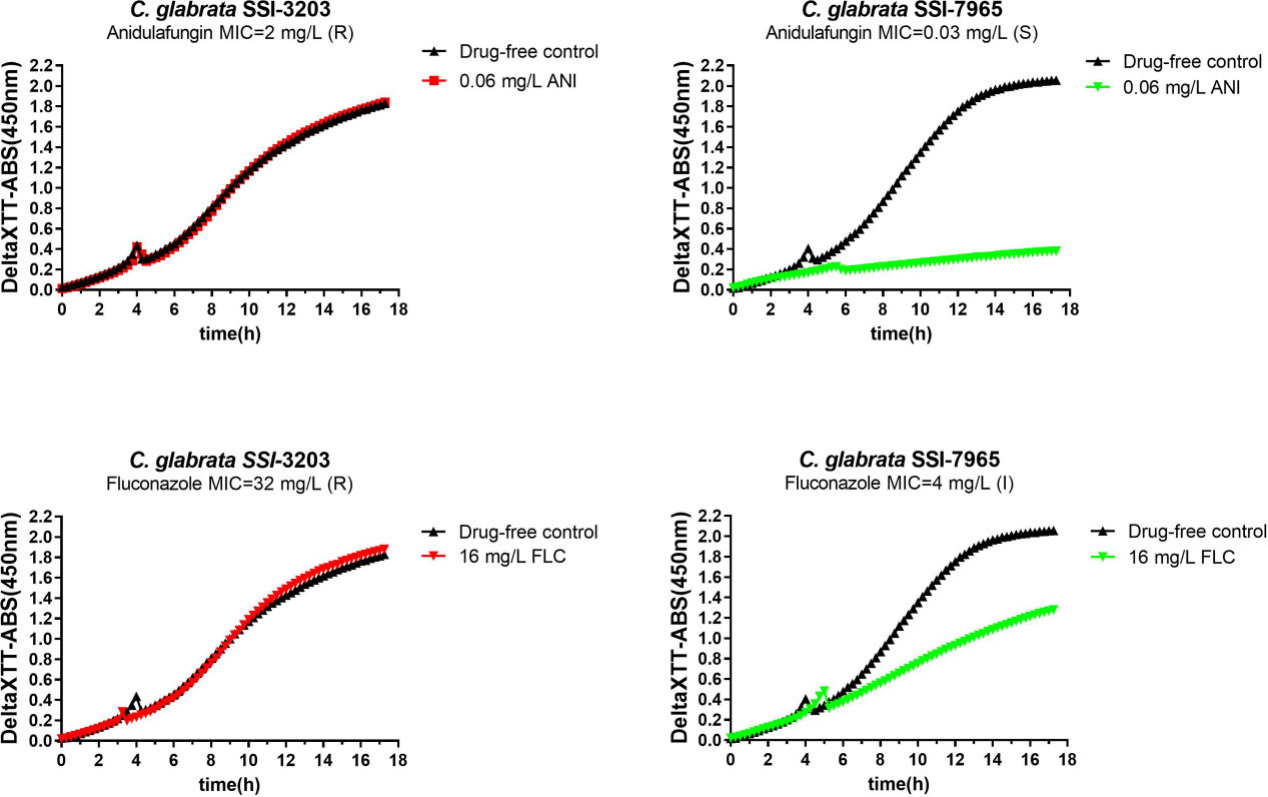
Metabolic curves generated with 100 mg/L XTT/0.78 μΜ menadione for an anidulafungin- and fluconazole-resistant *C. glabrata* isolate (SSI-3203) (left-hand panels) and an anidulafungin-susceptible and fluconazole-susceptible increased exposure *C. glabrata* isolate (SSI-7965) (right-hand panels). An increase around 4 h was observed for all of the curves, probably because of multiplication of *Candida* cells detected by the spectrophotometer. ANI, anidulafungin; FLC, fluconazole; S, susceptible; I, susceptible increased exposure; R, resistant. This figure appears in colour in the online version of *JAC* and in black and white in the print version of *JAC*.

XTT absorbance data for up to 18 h of incubation were generated using a spectrophotometer. All isolates converted XTT into its orange product, reaching an absorbance of >1.8 for the drug-free control (Figure 1). Metabolic curves were similar across the isolates in the absence of drug, with a linear increase in XTT absorbance up to 6 h, reaching an absorbance of 0.4, a sigmoid increase up to 12 h, reaching an absorbance of 1.6, and a slower increase up to 18 h, reaching a plateau at an absorbance of >2.0. In the presence of antifungal drugs, the metabolic curves of the resistant isolates were similar to the drug-free metabolic curves (Figure 1), whereas the metabolic curves of the susceptible isolates deviated over time. Thus, the metabolic curves of the susceptible isolates were almost flat in the presence of anidulafungin and increased more slowly compared with the drug-free metabolic curve in the presence of fluconazole.

### Optimization studies

The metabolic curves for anidulafungin-susceptible and anidulafungin-resistant isolates revealed no significant differences between the different XTT and menadione concentrations (data not shown). Metabolic curves for fluconazole-susceptible increased exposure and fluconazole-resistant isolates with drug added at 0, 2 and 4 h after inoculation showed that lower concentrations of both XTT and menadione with drug added at 0 h resulted in larger differences between drug-containing and drug-free metabolic curves for the susceptible isolate and smaller differences for the resistant isolate (data not shown). Thus, further experiments were conducted with 100 mg/L XTT + 0.78 μM menadione with 0.06 mg/L anidulafungin and 16 mg/L fluconazole.

### ROC curve analysis

**Figure 2.**
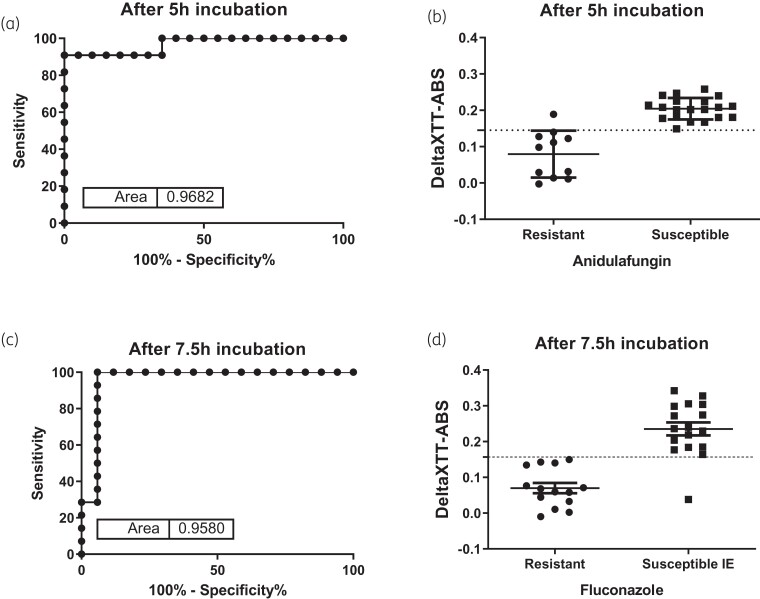
ROC curves and deltaXTT-ABS for resistant and susceptible *C. glabrata* isolates for anidulafungin (a and b) and fluconazole (c and d). Horizontal broken lines correspond to optimal deltaXTT-ABS of 0.145 and 0.157 for detecting resistance to anidulafungin and fluconazole, respectively. IE, increased exposure.

The AUC_ROC_ and the sensitivities/specificities at different timepoints are shown in Table 2. Timepoints <5 h for anidulafungin and <6.5 h for fluconazole resulted in specificities and sensitivities <65% (Table 2). The earliest timepoint with the highest sensitivity and specificity was 5 h for anidulafungin (AUC_ROC _= 0.968) and 7.5 h for fluconazole (AUC_ROC _= 0.958). For anidulafungin, the deltaXTT-ABS at 5 h of all resistant isolates, apart from one (strain SSI-7033), were lower than the deltaXTT-ABS of the susceptible isolates (mean ± SD 0.08 ± 0.07 versus 0.200 ± 0.03, respectively, *P *< 0.001) (Figure 2), with the highest likelihood ratio in ROC curve analysis found for a deltaXTT-ABS of <0.145 resulting in a sensitivity of 90.91% and a specificity of 100% (Table 2). For fluconazole, the deltaXTT-ABS at 7.5 h of resistant isolates were lower than the deltaXTT-ABS of all susceptible increased exposure isolates except one (strain SSI-3775) (mean ± SD 0.08 ± 0.05 versus 0.25 ± 0.06, respectively, *P *= 0.005) (Figure 2), with the highest likelihood ratio in ROC curve analysis found for a deltaXTT-ABS of <0.157, resulting in a sensitivity of 100% and a specificity of 94.12% (Table 2).

**Table 2.** Sensitivities and specificities of XTT method in separating resistant from susceptible/susceptible increased exposure strains at different timepoints

Drug, timepoint (h)	AUC_ROC_	Cut-off difference	Sensitivity (%)	Specificity (%)	Likelihood ratio
Anidulafungin					
4	0.845	<0.092	63.64	95	12.73
4.25	0.896	<0.114	81.82	95	16.38
4.5	0.864	<0.101	63.64	100	ND
4.75	0.923	<0.114	63.64	100	ND
**5**^a^	**0**.**968**	**<0**.**145**	**90**.**91**	**100**	**ND**
5.25	0.977	<0.166	90.91	100	ND
5.5^b^	0.986	<0.184	90.91	100	ND
5.75	0.986	<0.200	90.91	100	ND
6	0.986	<0.216	90.91	100	ND
Fluconazole					
6.5	0.878	<0.112	100	64.71	2.83
6.75	0.899	<0.127	100	64.71	2.83
7	0.932	<0.143	100	76.47	4.25
7.25	0.95	<0.150	100	88.24	8.5
**7**.**5**	**0**.**958**	**<0**.**157**	**100**	**94.12**	**17**
7.75	0.962	<0.171	100	94.12	17
8	0.958	<0.187	100	94.12	17

ND, not determined. Likelihood ratios [sensitivity/(100%–specificity)] cannot be calculated because specificity is 100%.

The earliest timepoint with highest sensitivity and specificity is shown in bold.

a A 100% sensitivity in distinguishing resistant strains from susceptible strains can be obtained using a deltaXTT-ABS <0.195. However, specificity at this deltaXTT-ABS was only 65% (data not shown).

b A 100% sensitivity in distinguishing resistant strains from susceptible strains can be obtained using a deltaXTT-ABS <0.225. However, specificity at this deltaXTT-ABS was 85% (data not shown).

## Discussion

A EUCAST E.DEF 7.3.2-based broth microdilution colorimetric assay was developed in the present study, enabling the detection of anidulafungin- and fluconazole-resistant *C. glabrata* isolates within 5 and 7.5 h of incubation with high sensitivity (91% and 100%) and specificity (100% and 94%), respectively. Since only one concentration and a drug-free control are required for detection of resistance, this assay could be incorporated in the 96-well EUCAST standard microplate and generate faster preliminary results while waiting for the final result after 24 h. Thus, detection of resistance is feasible within the 8 h working day, with a high sensitivity and specificity (>91%). Sensitivity was relatively lower for anidulafungin, due to one false-resistant isolate (delayed metabolic activity of the growth control), and specificity was relatively lower for fluconazole, due to one false-susceptible isolate (delayed metabolic activity in the drug-containing well). Prolonged incubation correctly classified these two isolates.

There is an increasing need to develop accurate and fast methods for detection of resistance. Over recent years molecular techniques have been used to detect resistance. These are PCR-based techniques that detect specific mutations in target genes that confer resistance to azoles and echinocandins. Echinocandin resistance is usually associated with specific mutations in the ‘hot-spot’ regions of *FKS1* and *FKS2* (for *C. glabrata* only) genes.^22^ Therefore, a PCR assay that detects most if not all mutations that are dominant could be designed based on local epidemiology. However, no such assays are commercially available yet and translation into level of resistance is dependent on the codon, the specific alteration and the species and thus expertise is required. For the azoles, the situation is more complex, as resistance is associated with multiple mechanisms, including mutations in *ERG11* and *ERG3* genes, overexpression of *ERG* genes, MDR or *Candida* drug resistance (CDR) efflux pumps, mutations in transcription factors or a combination of these mechanisms.^11,23,24^ Furthermore, they are difficult to use in the daily routine, they are expensive if multiple genes are targeted, skilled personnel are required and, perhaps most importantly, these molecular tests may detect resistance but not susceptibility unless the hot spots are sequenced in contrast to the rapid colorimetric testing presented here.

Flow cytometry has also been used to detect resistance to fluconazole within 1–2 h using serial drug dilutions and FUN-1 probes.^25,26^ Microfluidic cell-chip technology was used for testing susceptibility of *Candida* spp. to amphotericin B and fluconazole by differentiating live and dead cells.^27^ Although these approaches are very promising, they were applied to a small set of isolates not characterized molecularly and required serial dilutions of drugs, special equipment and expertise. MALDI-TOF MS has been used for antifungal susceptibility testing, with a sensitivity and specificity of 94% and 80% in detecting caspofungin-resistant *C. glabrata* isolates (CLSI MIC 0.5 to >8 mg/L) within 6 h using serial dilutions and caspofungin-resistant *C. albicans* isolates (CLSI MIC 1–4 mg/L) within 3 h with 1.6% very major errors.^14,15^ An imaging technique using porous aluminum oxide (PAO) was used to detect, within 1.5 h, microscopic changes in *Candida* isolates in an RPMI agar plate containing specific concentrations of antifungal drug, with high levels of agreement with EUCAST (>80%).^28^ However, both methods require rather expensive equipment, a separate set-up and certain expertise.

**Figure 3.**
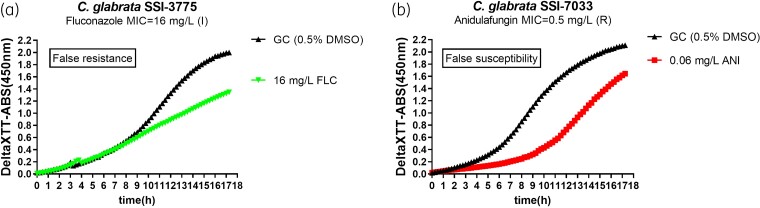
Examples of metabolic curves that showed false resistance to fluconazole, due to delayed metabolic activity of the drug-free growth control (a), or false susceptibility to anidulafungin, due to delayed metabolic activity in the drug-containing well (b). ANI, anidulafungin; FLC, fluconazole; GC, growth control; I, susceptible increased exposure; R, resistant. This figure appears in colour in the online version of *JAC* and in black and white in the print version of *JAC*.

The method described in the present study is cheap, with minimal hands-on time, requires no particular equipment and expertise and can be combined with the reference broth microdilution method in the same set-up. The only requirement is a microplate reader that can perform continuous measurements, which is present to most laboratories, due to its wide application. The method is very sensitive (100%) and specific (94%) in detecting fluconazole-resistant *C. glabrata* isolates within 7.5 h at a deltaXTT-ABS <0.157. Detection of echinocandin-resistant isolates can be achieved even earlier, after 5 h, with a sensitivity of 91% and a specificity of 100% at a deltaXTT-ABS <0.145. The method can detect resistance irrespective of the specific *FKS* mutation, providing that the mutation confers an MIC elevation. A higher sensitivity (100%) in detecting echinocandin resistance could be achieved by using a deltaXTT-ABS <0.195, although specificity was reduced (65%). The false results were due to the fact that one fluconazole-susceptible increased exposure isolate had a deltaXTT-ABS <0.157 (0.038) after 7.5 h, due to delayed metabolic activity of the growth control, and one anidulafungin-resistant isolate had a deltaXTT-ABS >0.145 (0.189) after 5 h, due to delayed metabolic activity in the drug-containing well (Figure 3). The false resistance for fluconazole could be overcome by introducing a cut-off of 0.324 for the drug-free control at 5.5 h in order to detect isolates with slow metabolic activity, whereas the false susceptibility for anidulafungin could be overcome by further incubation. More information on pathogen susceptibility and reduction of false results could be obtained by testing more than one concentration.

XTT has been used previously for early susceptibility testing of *Aspergillus* and Mucorales spp. within 6–8 h of incubation.^29,30^ For yeast, XTT was used to detect resistance, but almost 24 h was needed.^31^ In that study, the CLSI protocol was used with a lower inoculum size and RPMI with lower d-glucose. Unfortunately, XTT and menadione concentrations were not mentioned in order to conclude whether concentrations were sufficient to detect yeast metabolism, as XTT/menadione concentrations are important parameters for assessing metabolic activity. In the present study, XTT/menadione concentrations were optimized and in combination with the richer RPMI medium supplemented with 2% d-glucose and the higher inoculum size (0.5–2.5 × 10^5^ cfu/mL) of the EUCAST method we were able to detect metabolism early during incubation and distinguish resistant from susceptible isolates within one working day. The new method could be used for different *Candida* species and antifungal drugs after proper optimization of *in vitro* conditions.

In conclusion, the present paper describes a simple, cheap and fast phenotypic test that can detect resistance of *C. glabrata* to two classes of antifungal drugs. This opens the field for application to other drugs and species and possibly even directly from positive blood cultures, as long as a high fungal inoculum can be obtained free from other metabolically active cells. Fast phenotypic tests that correlate with reference methodologies of antifungal susceptibility testing can be useful tools in clinical laboratories for detecting antifungal resistance and guiding early appropriate antifungal therapy.
